# Trajectory modeling of cannabis use over 30 years identifies five unique longitudinal patterns

**DOI:** 10.1038/s41598-023-50376-x

**Published:** 2023-12-27

**Authors:** Amy R. Mahar, Michael P. Bancks, Stephen Sidney, Jared Reis, Gregory L. Kinney

**Affiliations:** 1 Department of Epidemiology, Harvard T.H. Chan School of Public Health, Boston, MA 02115 USA; 2 Department of Epidemiology and Prevention, Wake Forest University School of Medicine, Winston-Salem, NC USA; 3 Division of Research, Kaiser Permanente Northern California, Oakland, CA USA; 4 National Heart, Lung, and Blood Institute, Bethesda, MD USA; 5 Department of Epidemiology, Colorado School of Public Health, University of Colorado Anscshutz Medical Campus, Aurora, CO 80045 USA

**Keywords:** Epidemiology, Drug regulation, Health policy, Population screening

## Abstract

Cannabis is the most prevalently used psychoactive substance in the United States. Cannabis has conflicting federal and state legal status in the US, however medical and recreational cannabis use are increasing. When assessing health outcomes, cannabis use classification has been modeled largely as current use status (never/former/current) or cumulative use (joint-years). These methods do not describe longitudinal patterns of use which may have unique relationships with health outcomes. We used cannabis use data spanning 30 years from the Coronary Artery Risk Development in Young Adults Cohort (CARDIA) to create trajectories of current cannabis use during young and middle adulthood. We identified 5 unique patterns of the probability of cannabis use during young and middle adulthood in the CARDIA Cohort. To support the cannabis probability trajectories, we qualitatively examined cumulative cannabis use as joint-years for each trajectory group. Trajectory group 5 had high probability of consistent cannabis use (0.8–0.9% probability of use) and had the highest number of joint-years (0.6 +/− 0.4). Trajectory group 1 who had a lower probability of cannabis use (0.05–0% probability of use) with the lowest number of joint-years (0.1 +/− 0.1).

## Introduction

Investigating the health consequences of cannabis use requires characterizing cannabis use in a manner relevant to the research question. Lifetime cannabis use has been modeled in a variety of ways including current status (never/former/current) and cumulative use (joint-years), but these approaches cannot characterize longitudinal patterns of varying use. Few studies have followed cannabis users for an extended time (≥ 20 years) before and after initiation of use. Two studies repeatedly assessed cannabis use across 20 years beginning from adolescence, but each study summarized cumulative use and did not characterize longitudinal patterns of use^1,2^.

The Victorian Adolescent Healthcare Study (VAHCS) found that cannabis use was present at least once in 62% of their participants and that current use was most prevalent at age 20 and declined to 5% by age 35. VAHCS then examined health outcomes based on dividing the cohort into groups based on the age of cannabis initiation and found that early adolescent cannabis use was associated with cannabis dependence and increased use of other substances, and with harms to academic performance and mental health. The Dunedin Study examined lifetime cannabis use by summarizing cannabis use over 20 years using joint-years of exposure and found no significant health effects by age 38 apart from increased periodontal disease. Both studies discuss potential biological associations between cannabis as an exposure and health outcomes, but it is likely that patterns of use that change over time will result in a pattern of risk that also varies over time. We examined 9 observations of past 30 days cannabis use covering 30 years of life using data from the Coronary Artery Risk Development in Young Adults (CARDIA) study collected at concurrent study visits to develop cannabis use trajectories describing patterns of use from young adulthood to middle age.

## Methods

CARDIA participants were aged 18–30 years in 1985–1986 (enrollment) and have been invited to participate in 8 follow-up examinations over 30 years. Demographics and the study design of the cohort have been published previously, and Institutional Review Boards at each field center approved CARDIA study protocols^3^. Participants provided written informed consent for each CARDIA exam they participated in. Participant response to the question “During the past 30 days, on how many days did you use marijuana?” on the substance use questionnaire was used to assess the probability of current cannabis use. Current use at each study visit was defined as “yes” if reported use of cannabis ≥ 1 day of use over the last 30 days and “no” if reported as 0 days in the last 30 days. CARDIA participants could decline to answer any portion of the questionnaire. At least three data points are needed to determine a trajectory (two data points indicate a change in direction, not a trajectory) and participants who had fewer than three cannabis responses were excluded. If participants missed a study visit, they were still invited back to participate in future follow up visits.

To identify trajectories, we used discrete mixture modeling techniques designed to identify sub-populations of participants with similar characteristics longitudinally. We used the Bernoulli distribution based logistic model implemented by Proc Traj in the SAS system to identify trajectories of cannabis use over time. We utilized the Bernoulli because of the binary variable (yes/no). This approach generates a probability model of trajectory group membership which describes the distribution of the trajectories in the population accounting for uncertainty in membership. It also assigns group membership to participants assuming perfect classification where a participant is assigned to a group based on the highest probability of membership but does not account for uncertainty in assignment. This potential error in individual participants’ group assignment is addressed in the approach by ensuring that the correspondence between the average of the posterior probability of group membership and membership assignment exceeds a threshold of 0.7^4^.

The count of trajectories is examined across a range chosen by the investigator and a final count is determined based on model fit and terms describing the shape of the trajectory from linear to higher level terms. We tested models from three to six trajectories and terms for linear, quadratic, cubic, quartic and quintic order in each model. We chose the model with the lowest Bayesian information criterion (BIC).

## Results

There were 5115 participants in the CARDIA study at the baseline exam and 4668 participants provided cannabis use data during at least 3 of the 9 time points. The participants who did not have 3 or more cannabis data points (n = 477) tended to be black (68.5%) versus white, however gender was approximately equal (49.9% male) and geographically they were roughly evenly distributed across study centers (22.2% in Birmingham, 24% in Chicago, 28% in Minneapolis, and 25% in Oakland).

**Figure 1:**
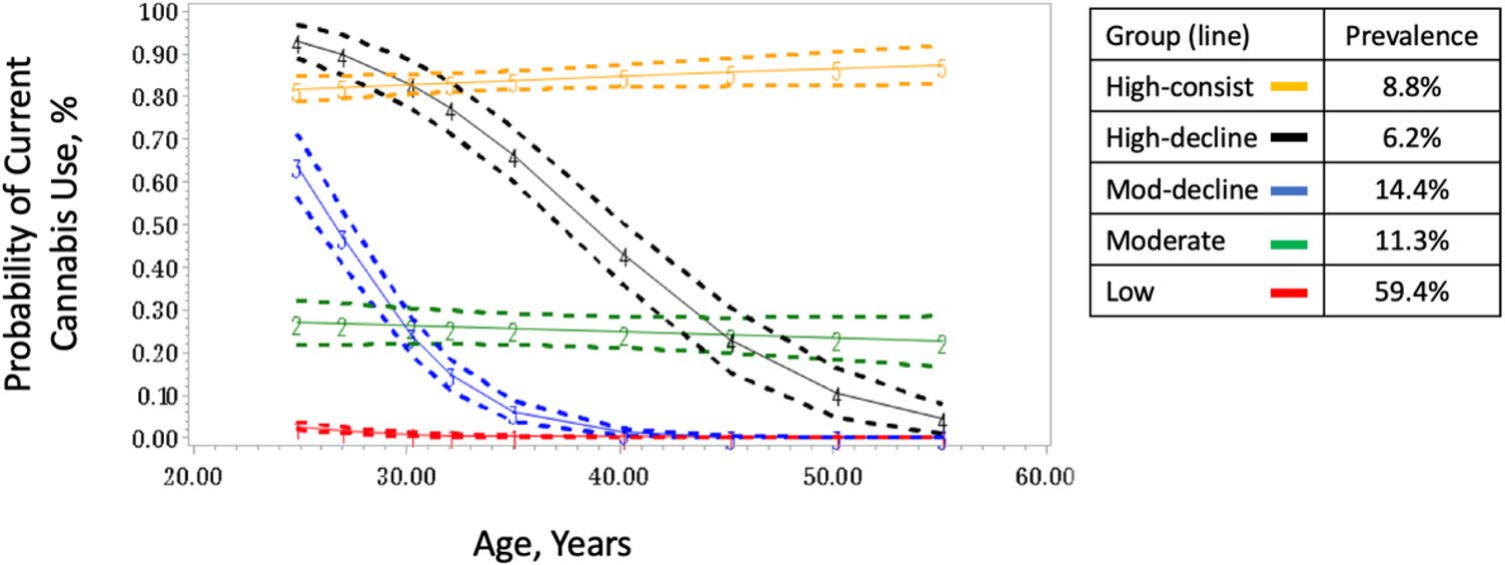
Cannabis use trajectories in CARDIA.

Five trajectory groups, each with linear order best fit the data for probability of current cannabis use, generating the largest improvement in BIC and qualitative assessment of group size and pattern (Fig. 1). Group 1 (59.4% prevalence of the study population assigned to this group) had a zero to very low probability of cannabis use across exams thus named ‘Low’. Group 2 (11.3% prevalence) had a 20%-30% probability of current cannabis use across exams, thus named ‘Moderate’. Group 3 (14.4% prevalence) was named ‘Mod-decline’ due to having a moderately high, 60–70% probability of current cannabis use at age 25, which decreased to a probability of zero by age 40 and beyond. Group 4 (6.2% prevalence) had a very high probability of current use at age 25, 90–100% probability, which decreased over the next 30 years to a probability of near zero by age 55, named ‘High-decline’. Group 5 (8.8% prevalence) had a probability of current cannabis use ≥ 80% during the 30 years of observation and was named ‘High-consistent’.

Figure 1 shows the most probable cannabis use trajectories and the mean probabilities of group membership in 4668 CARDIA participants over 30 years of follow-up. Group Percents represent trajectory creation accounting for uncertainty. The X axis represents participant age and the Y axis represents the probability of past 30 day use. Group 1 was named low, Group 2 named moderate, Group 3 named mod-decline, Group 4 named high-decline, and Group 5 named high-consist.

Trajectory group assignment for individuals was consistent with trajectory group creation. The largest discrepancy occurred for Group 1, ‘Low’, where 64.2% of the population generated that trajectory, but 59.4% of the population were assigned to it due to that trajectory describing their most probable trajectory of use. Table 1 illustrates demographic characteristics for each trajectory group at baseline or at baseline and at the 10 years follow-up visit for demographics that change over time. The trajectories captured the expected accumulation of cannabis exposure in a predictable way where Groups 4 (high-decline) and 5 (high- consistent) accumulated the largest average number of joint-years (2.7 ± 2.4 and 3.7 ± 2.8 respectively at year 10) and Group 1 (low) accumulated very little exposure (0.1 ± 0.1 at year 10). Group 1 (Low) was more likely to be female and consistently abstain from smoking cigarettes and regular alcohol use. Of note, unemployment was not strikingly different at baseline or 10-year follow-up visits across trajectory groups, though Group 1 was the least likely to be unemployed at the 10-year follow-up visit.

**Table 1:** Participant characteristics at year 0 (baseline) and at year 10 years follow-up by cannabis trajectory group.

Trajectory group n (%)	Group 1, low	Group 2, moderate	Group 3, moderate-decline	Group 4, high-decline	Group 5, high-consistent
	n = 2960 (63)	n = 374 (8)	n = 637 (14)	n = 265 (6)	n = 432 (9)
Year 0					
Joint Years at year 0^a^	0.1 (0.1)	0.2 (0.2)	0.4 (0.3)	0.6 (0.4)	0.6 (0.4)
BMI^a^					
Exam year 0	24.5 (5.2)	24.4 (5.2)	24.4 (4.5)	24.5 (4.9)	24.7 (4.8)
Age^a^					
Exam year 0	24.8 (3.7)	25.2 (3.5)	25.0 (3.3)	26.0 (3.4)	24.9 (3.6)
Race					
Black	1433 (48.4)	210 (56.2)	321 (50.4)	139 (52.5)	228 (52.8)
White	1527 (51.6)	164 (43.9)	316 (49.6)	126 (47.6)	204 (47.2)
Sex					
Male	1149 (38.8)	166 (44.4)	343 (53.9)	156 (58.9)	290 (67.1)
Female	1811 (61.2)	208 (55.6)	294 (46.2)	109 (41.1)	142 (32.9)
Center					
Birmingham	785 (26.5)	60 (16.0)	121 (19.0)	47 (17.7)	49 (11.3)
Chicago	645 (21.8)	72 (19.3)	164 (25.8)	57 (21.5)	62 (14.4)
Minneapolis	723 (24.4)	120 (32.1)	170 (26.7)	88 (33.2)	153 (35.4)
Oakland	767 (25.9)	114 (30.5)	174 (27.3)	69 (26.0)	159 (36.8)
Unemployed at year 0					
No	2163 (73.1)	247 (66.0)	428 (67.2)	172 (64.9)	290 (67.1)
Yes	789 (26.7)	125 (33.4)	208 (32.7)	93 (35.1)	141 (32.6)
Missing	8 (0.2)	2 (0.5)	1 (0.2)	0 (0.0)	1 (0.2)
Cigarette smoking status at year 0					
Never	2007 (67.8)	135 (36.1)	258 (40.5)	92 (34.7)	147 (34.0)
Former	339 (11.5)	75 (20.1)	96 (15.1)	41 (15.5)	75 (17.4)
Current	600 (20.3)	159 (42.5)	278 (43.6)	131 (49.4)	204 (47.2)
Missing	14 (0.5)	5 (1.3)	5 (0.8)	1 (0.4)	6 (1.4)
ETOH at year 0					
0 drinks/d	1465 (31.4)	115 (2.5)	110 (2.4)	40 (0.8)	85 (1.8)
0–1 drinks/d	1101 (23.6)	184 (3.9)	300 (6.4)	122 (2.6)	164 (3.5)
> 1 drinks/d	378 (8.1)	74 (1.6)	226 (4.8)	102 (2.2)	183 (3.9)
Missing	16 (0.5)	1 (0.3)	1 (0.2)	1 (0.4)	0 (0)
Year 10 trajectory groups N (%)	2614 (62.8)	344 (8.3)	541 (13.0)	225 (5.4)	353 (8.5)
Joint years at year 10	0.1 (0.1)	0.7 (0.9)	0.8 (0.9)	2.7 (2.4)	3.7 (2.8)
BMI^a^					
Exam year 10	27.6 (6.8)	27.2 (6.3)	27.7 (6.1)	27.3 (5.9)	27.1 (5.7)
Age^a^					
Exam year 10	34.8 (3.8)	35.2 (3.6)	35.2 (3.3)	36.1 (3.3)	35.0 (3.7)
Unemployed at year 10					
No	2284 (77.2)	275 (73.5)	450 (70.6)	179 (67.6)	295 (68.3)
Yes	205 (6.9)	59 (15.8)	76 (11.9)	39 (14.7)	49 (11.3)
Missing	4 (0.1)	0 (0.0)	1 (0.2)	0 (0.0)	0 (0.0)
Cigarette smoking status at year 10					
Never	1734 (58.6)	118 (31.6)	230 (36.1)	75 (28.3)	114 (26.4)
Former	388 (13.1)	64 (17.1)	101 (15.9)	32 (12.1)	61 (14.1)
Current	376 (12.7)	154 (41.2)	196 (30.8)	108 (40.8)	167 (38.7)
Missing	462 (15.6)	38 (10.2)	110 (17.3)	50 (19.9)	90 (20.8)
ETOH at exam year 10					
0 drinks/d	1416 (47.8)	100 (26.7)	210 (33.0)	46 (17.4)	76 (17.6)
0–1 drinks/d	838 (28.3)	131 (35.0)	193 (30.3)	76 (28.7)	122 (28.2)
> 1 drinks/d	236 (8.0)	103 (27.5)	124 (19.5)	96 (36.2)	143 (33.1)
Missing	470 (15.9)	40 (10.7)	110 (17.3)	47 (17.7)	91 (21.1)

Data are presented as n (%) except where otherwise indicated ^a^Data are presented as mean (SD). Trajectories are identified including uncertainty as shown using error bars in Fig. 1 but trajectory membership is assigned based on the most probable trajectory membership causing the group membership percentage to deviate from the estimated trajectory definition Group Percents.

## Discussion

CARDIA cannabis use data has been reported previously as it relates to outcomes relevant to the cohort. Null associations were reported between cannabis use (joint-years and past 30-day use) and abdominal adiposity^5^, incident diabetes^6^ and CVD risk^7^. However, Auer et al.^8^ showed that lower verbal memory in adulthood was associated with cumulative cannabis exposure as well as current use. VAHCS and The Dunedin Study identified significant lifetime health outcomes as the result of cannabis use measured similarly, though several outcomes were associated with current use of cannabis where the outcome was temporally relevant, for instance educational attainment for current users. Our study is the first application of group-based trajectory modeling of cannabis use in CARDIA. Cannabis use was assessed via questionnaire response at intervals ranging from 2 to 5 years and does not capture year-to-year variation in cannabis use. The CARDIA study design has many strengths, including the long duration of observation during a period of life when cannabis use is most frequently reported. However, we must acknowledge limitations of the cannabis data collection in CARDIA which do not include information on within-day frequency of use, amount of cannabis per use, or route of administration.

Cannabis use is typically assessed as either a current exposure or as an accumulation of exposure like assessment of tobacco cigarette use. This approach may persist due to the assumption that smoking is the most common mode of cannabis use and that this should result in similar outcomes compared to tobacco use. This assumption may need reconsideration as some literature suggests cannabis use may not impact measures of pulmonary function as assessed using spirometry in similar magnitude as tobacco use. This may be due to the volume of cannabis plant ingested or to the increasing frequency of cannabis exposure that is not due to pyrolysis of the cannabis flower. Indeed, vaping and other low temperature approaches are used currently to mitigate the generation of harmful pyrolysis products for both cannabis and tobacco. Ingestion of cannabis via the lungs has been shown to cause symptoms of lung irritation however these symptoms (cough, wheeze, sputum production) appear to be transient suggesting that the acute effects of cannabis ingestion may be important for *some* health outcomes but not for others. To understand health outcomes related to cannabis use it may be necessary to understand other aspects of that use that go beyond those obtained using assumptions based on cigarette smoking and to that end we propose cannabis use trajectories. Trajectories may indicate patterns of use over the life course that are obscured by relying on either current use or the total accumulation of exposure via joint-years and add to statistical models of health outcomes. Our results identify and describe distinct patterns of cannabis use spanning young and middle adulthood, which were not previously characterized. Assessment of cannabis use trajectories provides a combined qualitative (pattern of use) and quantitative (probability of use) understanding of longitudinal cannabis use as an exposure and how trajectory of use may impact health outcomes differently. Future work will assess whether these effects are independent and whether these patterns are generalizable to other populations.

## Data Availability

The data analyzed for this study are available through the CARDIA Coordinating Center: https://www.cardia.dopm.uab.edu/. This application process will include scientific review, institutional review and approval, and the completion of a data and material distribution agreement. CARDIA data are also publicly available via application to the NIH: https://biolincc.nhlbi.nih.gov/studies/cardia/.
